# Designing Antioxidant and Antimicrobial Polyethylene Films with Bioactive Compounds/Clay Nanohybrids for Potential Packaging Applications

**DOI:** 10.3390/molecules28072945

**Published:** 2023-03-25

**Authors:** Konstantinos Safakas, Iro Giotopoulou, Archontoula Giannakopoulou, Katerina Katerinopoulou, Georgia C. Lainioti, Haralambos Stamatis, Nektaria-Marianthi Barkoula, Athanasios Ladavos

**Affiliations:** 1 Department of Food Science & Technology, University of Patras, GR-30100 Agrinio, Greece; 2 Department of Materials Science and Engineering, University of Ioannina, GR-45110 Ioannina, Greece; 3 Department of Biological Applications and Technology, University of Ioannina, GR-45110 Ioannina, Greece

**Keywords:** bioactive compounds, carvacrol, thymol, olive leaf extract, clay, low-density polyethylene, antioxidant activity, antimicrobial activity

## Abstract

In the present work, direct incorporation of bioactive compounds onto the surface and interlayer of nanoclays before their incorporation into the final polymeric film was conducted, based on a green methodology developed by our group that is compatible with food packaging. This will lead to the higher thermal stability and the significant reduction of the loss of activity of the active ingredients during packaging configuration. On this basis, the essential oil (EO) components carvacrol (C), thymol (T) as well as olive leaf extract (OLE), which is used for the first time, were incorporated onto organo-modified montmorillonite (O) or inorganic bentonite (B) through the evaporation/adsorption method. The prepared bioactive nanocarriers were further mixed with low-density polyethylene (LDPE), via melt compounding, in order to prepare films for potential use as fresh fruit and vegetable packaging material. Characterization of the bioactive nanocarriers and films were performed through XRD, TGA, tensile, antimicrobial and antioxidant tests. Films with organically modified montmorillonite loaded with carvacrol (OC), thymol (OT) and olive leaf extract (OOLE) at 5% wt. showed better results in terms of mechanical properties. The films with polyethylene and organically modified montmorillonite loaded with carvacrol or thymol at 20% wt. (PE_OC20 and PE_OT20), as well as with olive leaf extract at 5 or 10 %wt., clay:bioactive substance ratio 1:0.5 and 10% compatibilizer (PE_OOLE5_MA10 and PE_OOLE10_MA10) exhibited the highest antioxidant activity. The resulting films displayed outstanding antimicrobial properties against Gram-negative *Escherichia coli* (*E. coli*) with the best results appearing in the films with 10% OC and OT.

## 1. Introduction

The food industry is a very dynamic sector that is consistently changing, as motivated by consumers’ preferences and health anxieties. Packaging is vital, since it ensures safe transportation and storage of food and protects it from contamination, spills and atmospheric conditions [1,2]. The increasing world population and globalization have led to increasing demands in today’s society for novel food packaging solutions which may interact with the food product providing enhanced sensory and/or safety properties [3]. Active packaging, intelligent packaging and bioactive packaging constitute the major innovations in the field of packaging technology, and their action concerns food product shelf life extension, quality enhancement and freshness regulation [4].

Bioactive compounds and their composites are widely applied in food packaging [5,6]. Their incorporation into the packaging wall can impart antioxidant and antimicrobial properties leading to so-called bioactive food packaging, the main characteristic of which is the controlled release of the bioactive compounds during food storage prior to its consumption [7,8]. The bioactive packaging technology is applied through (i) the employment of biodegradable packaging materials for functional or bioactive components’ release (integration and controlled release) [9,10,11], (ii) the bioactive ingredients’ encapsulation into the food products or packaging materials (micro- and nanoencapsulation) [12,13,14] and (iii) the introduction of packaging materials with the ability to transform some food components through enzyme activity (enzymatic packaging) [15,16].

During recent years, food packaging research has been focused on an innovative trend related to the combination of natural bioactive compounds with nanoparticles. The most common bioactive compounds used are essential oils (EO). Carvacrol (5-isopropyl-2-methylphenol), together with its isomer thymol (2-isopropyl-5-methylphenol), are natural phenolic compounds and major components of oregano and thyme essential oils, responsible for their biological activity. They have antibacterial, antifungal and antiseptic activities [17,18] and are used as food preservatives. A wide range of EOs have been used as additives directly in food or edible/biodegradable food packaging due to their high antibacterial and antioxidant effects. This may lead to the enhancement of shelf life and quality characteristics of food, as well as the protection of consumers from oxidative and bacterial deterioration effects. For the incorporation of additives, such as EOs in a polymer matrix, the melt mixing technique in screw extruders is usually applied in industry. However, their volatile nature is an inhibitory factor for their use in such processes because this would lead to their rapid loss via evaporation [19,20].

On this basis, nanoencapsulation of bioactive compounds has been proposed as a promising technique in order to improve stability and to solve the drawbacks of their direct embedding in polymers [21]. Therefore, the adsorption of EOs onto an inorganic porous material could provide the required controlled release and protection in the polymer process [22,23]. Novel bio-nanocomposites, such as nanoclay supporting active molecules may be integrated into the packaging materials, providing an emerging technique to produce new packaging with various functionalities. Polymer–clay nanocomposites are an alternative to conventional polymers due to their nanoscale dispersion with enhanced mechanical, thermal and barrier properties of the polymer films [24,25]. The mixtures of natural compounds with antimicrobial and antioxidant properties with polymers to improve functional properties and extend food shelf life show an increasing interest by the food sector [26,27,28]. Clay minerals have been extensively used as carriers of bioactive compounds for a variety of applications, including food packaging and in agricultural fields. Montmorillonite (Mt) is the type of clay most extensively investigated for such applications. It is a hydrated alumina-silicate multilayer clay, which comprises a sandwich structure of an octahedral aluminum hydroxide layer shared between two tetrahedral silica layers. The exchangeable Na^+^ and Ca^++^ cations [29] balance the surface negative charges. Mt can be easily organo-modified with the cation exchange of various surfactants. The high aspect ratio of Mt platelets, however, generates a significant enhancement in polymer nanocomposite properties at low loadings when they are highly dispersed. For the improvement of the dispersion of Mt particles into the polymer matrices, a compatibilizer is often required [30]. Moreover, as most of the polymers are organophilic, organically modified layered silicates are employed to obtain a better affinity between the filler and the matrix.

Significant research has been conducted towards the incorporation of essential oil constituents thymol and carvacrol, loaded in nanocomposites, into polymers to enhance antimicrobial and antioxidant functionality [31,32,33]. In the literature, the incorporation of EO on inorganic nanomaterials is reported with the use of encapsulation [34,35], impregnation [23,36] or adsorption with the use of organic solvents, such as acetone [37] or heptane [38], which may lead to possible residues of solvents in the final products that are present throughout the packaging material. In our lab, a green methodology was recently developed for the direct incorporation of EO components onto clays through the evaporation/adsorption method, without the use of organic solvents.

The aim of this work is the use of the unmodified montmorillonite (bentonite) as well as the organo-modified montmorillonite (O) as carriers of active volatile compounds, carvacrol (C) and thymol (T), as well as olive leaf extract (OLE), to produce active clay nanoparticles through a green methodology. Specifically, the incorporation of carvacrol and thymol into the phyllosilicate clays was performed through the adsorption method, without the use of any solvent, whereas for the incorporation of the olive leaf extract, which is used for the first time, the solution method was applied. The bioactive compounds loaded into nanoclay were subsequently incorporated into LDPE, leading to the production of films with controlled antimicrobial and antioxidant performance, in terms of food quality. The prepared bioactive nanocarriers and the bioactive nanocomposite films were characterized via XRD, TGA, tensile, antioxidant and antimicrobial tests.

To the best of our knowledge, little research has directly addressed to the incorporation of organo-modified montmorillonite or inorganic bentonite loaded with thymol and carvacrol in LDPE-based films for the preparation of active nanocomposites with antimicrobial and antioxidant properties [25,39,40,41], whereas olive leaf extract is reported for the first time. The present work could provide substantial knowledge and fill a gap in the preparation of novel nanocomposite films with antibacterial and antioxidant properties which would be potentially used as active food packaging materials.

## 2. Results

### 2.1. Characterization of the Prepared Bioactive Nanocarriers

As it was mentioned in the Introduction, the main aim of this study is the development of flexible films with antioxidant and antimicrobial properties for food packaging application. On this basis, the adsorption of bioactive compounds to the interlayer/surface of the clays was initially conducted to maximize the incorporation degree and the controlled release of bioactive compounds. Thus, the first step was the adsorption of C, T and OLE onto O and B. The clay:bioactive compound ratios (*r*) examined in the present work were 1:0.01, 1:0.1, 1:0.5, 1:0.7, 1:0.8, 1:0.9 and 1:1, as shown in Table 1. The ratio *r* varied depending on the clay used and the bioactive compound loaded in each clay.

#### 2.1.1. XRD Results

**Figure 1:**
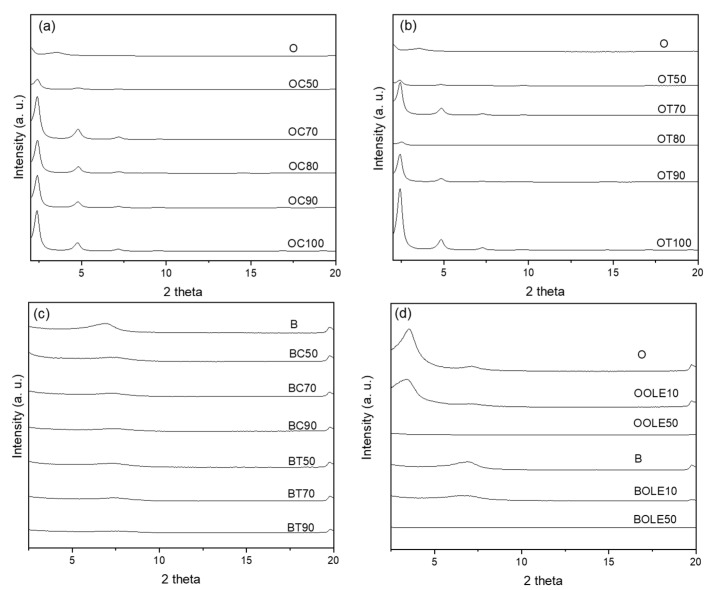
XRD patterns of bioactive nanocarriers: (**a**) carvacrol-loaded organically modified montmorillonite (OC), (**b**) thymol-loaded organically modified montmorillonite (OT), (**c**) carvacrol- and thymol-loaded bentonite (BC, BT) and (**d**) olive leaf extract-loaded organically modified montmorillonite (OOLE) and bentonite (BOLE).

Figure 1 shows the XRD patterns of all the prepared samples, the organically modified montmorillonite loaded with carvacrol (OC) (Figure 1a), thymol (OT) (Figure 1b) and OLE (OOLE) (Figure 1d) and bentonite loaded with carvacrol (BC), thymol (BT) (Figure 1c) and OLE (BOLE) (Figure 1d). The plots of the neat nanoclays are also reported for comparison reasons. The *d*_001_ values for all bioactive nanocarriers were calculated and are presented in Table 1. The XRD pattern of the organo-modified montmorillonite (O) indicated a diffraction peak at 2θ of about 3.54°, corresponding to *d*_001_ of 25.0 A˚, characteristic of montmorillonite clay modified with a dialkyl group [42]. As shown from the XRD patterns of Figure 1a, the peak of the organically modified clay (O) at 3.54° shifted to lower 2θ values, between 2.37 and 2.65°, after the incorporation of the bioactive compounds, carvacrol (C) or thymol (T) from *r* = 1:0.1 to 1:1. The 001 reflections (Figure 1) and the calculated *d*_001_ values (Table 1) of the OC and OT nanocomposites showed an obvious increase in the basal spacing after C or T addition in comparison with the neat O sample. More specifically, the *d*_001_ spacing was 25 Å for the neat O sample, whereas when carvacrol was added the basal spacing, it was found to range from 25.7 Å for *r* = 1:0.01 of carvacrol, to 37.3 Å for *r* = 1:1 of carvacrol. In the case of OT nanocomposites, the addition of thymol also led to an increase in the basal spacing from 25.8 Å for *r* = 1:0.01, to 36.7 Å for *r* = 1:1. In general, it was observed that as the percentage of the bioactive compound in the nanocarrier increases, the distance *d* between the clay sheets widens. The shift of the peak to the left and at lower 2θ degrees confirms the incorporation of the bioactive compounds in the nanocarrier. The increase in the distance *d* between the clay sheets indicates the encapsulation of the bioactive compound molecules between the clay lamellae.

Furthermore, from the results of Table 1, it is obvious that there was no considerable change in the interplanar *d*-spacing of the bioactive nanocarriers from *r* = 1:0.5 to *r* = 1:1 for both carvacrol and thymol, indicating a probable bioactive compound saturation between clay lamellae at these contents.

In the case of bentonite, the characteristic reflection 001 occurs at 2θ = 6.82°. However, with the adsorption of the bioactive compounds, as well as the increase in their content in the different samples, there is a shift of the peak to higher 2θ values (7.10–7.34°) while its intensity weakens, due to the loss of adsorbed water. The above result is an indication that the bioactive compounds do not enter the interlayer region of the bentonite sheets but are mainly retained on the outer surface of the clay particles. The 001 reflections (Figure 1) and the calculated *d*_001_ values (Table 1) of the BC and BT nanocomposites showed a decrease in the basal spacing after C or T addition in comparison with the neat bentonite sample. Specifically, the *d*_001_ spacing was 13 Å for neat bentonite, whereas the addition of carvacrol led to values from 12.3 to 12.0 Å for *r* = 1:0.5 to *r* = 1:0.9, and the addition of thymol gave values from 12.5 to 12.2 for *r* = 1:0.5 to *r* = 1:0.9. Therefore, bioactive compounds’ adsorption onto the bentonite substrate did not increase the basal spacing, indicating the adsorption of C and T on the surface and not in the interlayer of the clay nanocarrier.

Concerning the nanocarriers where olive leaf extract was embedded, regardless of the type of carrier (organically modified or inorganic), in a small proportion the peak position corresponded to a small increase in the distance *d*_001_. With the increase in the ratio, the 001 reflection of the nanocarriers did not occur, as shown in Figure 1d. This result is indicative of exfoliated clay structure.

Consequently, the bioactive compounds’ adsorption in the bentonite nanocarrier did not increase the basal spacing of bentonite layers, indicating that the adsorption of C, T and OLE took place at different surface sites. On the other hand, the adsorption of bioactive compounds onto the O carrier led to a homogenous opening of the clay layers and the adsorption of C, T and OLE in the interlayer space.

Moreover, the BET specific surface area of inorganic bentonite is 59 m^2^/g, whereas of the organo-modified montmorillonite it is 194 m^2^/g [43]. The higher surface area of organically modified montmorillonite compared to bentonite is an indicative factor for the lower adsorption of bioactive compounds onto the inorganic bentonite in comparison to the organo-modified montmorillonite.

#### 2.1.2. TGA Results

**Figure 2:**
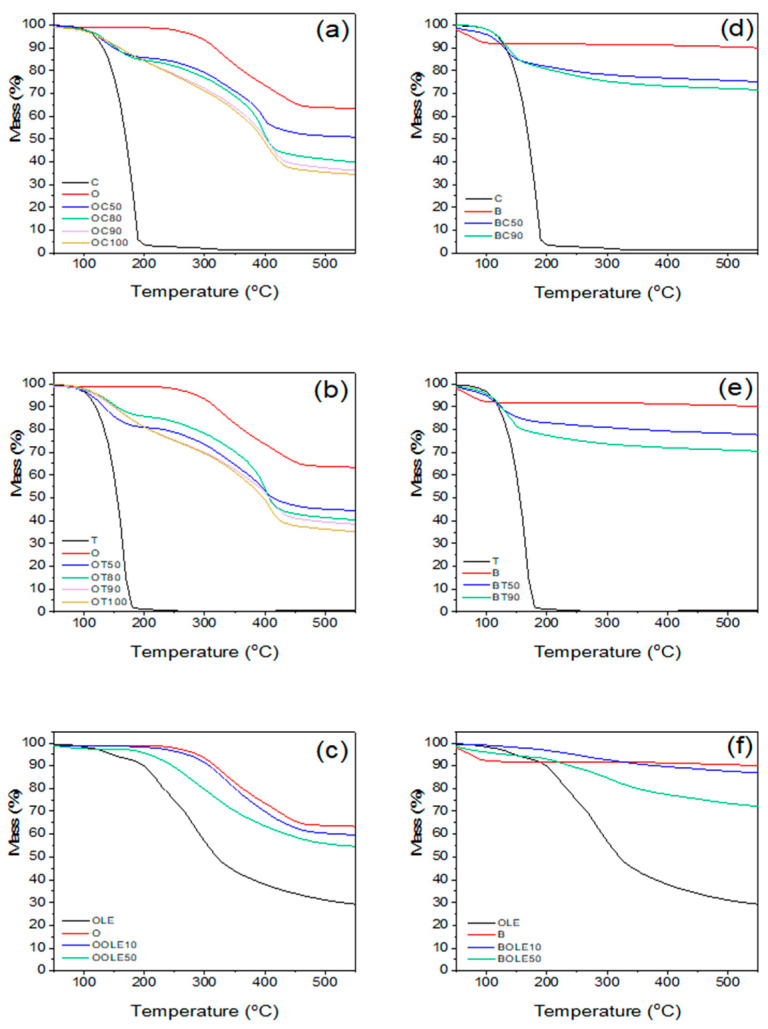
TGA profiles of neat clay and bioactive nanocarriers: (**a**) carvacrol-loaded organically modified montmorillonite (OC), (**b**) thymol-loaded organically modified montmorillonite (OT), (**c**) olive leaf extract-loaded organically modified montmorillonite (OOLE), (**d**) carvacrol-loaded bentonite (BC) (**e**) thymol-loaded bentonite (BT) and (**f**) olive leaf extract-loaded bentonite (BOLE).

Additionally, the clays loaded with the bioactive compounds prepared in the present work were further analyzed through thermogravimetric analysis (TGA). The study of thermal analysis of bioactive nanocarriers is very important as it will provide insights into how they behave as heat flows. The data from thermal stability analysis are presented in Table 1, while TGA profiles are graphically presented in Figure 2.

Among the nanocarriers with incorporated C and T, those with *r* = 1:0.5 and above were chosen to be studied, whereas the ratios *r* = 1:0.1 and 1:0.5 were studied in the case of OLE. The decomposition temperatures (*T*_20_) reported in Table 1 include the 20% mass loss temperature. The bioactive substance content (% wt.) on the nanocarrier has been calculated from TGA graphs. Regarding the thermal stability of the nanocarriers, TGA measurements confirm the protective effect of the adsorption of the bioactive substance within the clay. The studied C and T nanocarriers with O indicate a two-step decomposition temperature. The first decomposition temperature, above 100 °C, corresponds to the release of C or T molecules adsorbed in the O interlayer space, and the second decomposition temperature to the decomposition of the organic modifier of the O carrier. The adsorption of the bioactive substance, (C) or (T), on the organically modified clay (O) results in an increase in its complete loss temperature by 37–50 °C compared to the pure bioactive substance. In the case of carvacrol and thymol nanocarriers with bentonite, one decomposition step was observed at ~150 °C, which corresponds to the decomposition of the bioactive compounds. The total bioactive substance content in the clays as determined by TGA showed that the bioactive substances’ concentration varied at 14–29% wt. for C, 19–26% wt. for T and 4–9% wt. in the case of OLE when organically modified montmorillonite was used as nanocarrier. In the case of B, the percentages were lower in most cases, except for BOLE50, which showed 18% incorporation of OLE into the B. It is evident from the results that encapsulation in O is beneficial for T or C while encapsulation in B is beneficial for OLE. This behavior is owed to the fact that the adsorption process of thymol and carvacrol molecules takes place on the external surface of bentonite by hydrogen bonds between thymol or carvacrol OH groups and OH groups of bentonite that exist on the surface. However, in the case of organo-modified Mt, the adsorption takes place not only on the external surface but mainly in the interlayer space due to the organophilic environment generated by the presence of surfactant compounds. The opposite behavior of olive leaf extract (higher adsorption on inorganic bentonite) is due to the hydrophilic nature of OLE.

The contents indicated that >50% of the initial bioactive substance content has been incorporated into the clays. The incorporation of carvacrol and thymol into nanoclays showed a significant enhancement in thermal stability. The results are in accordance with the results reported by Krepker et al. [44] showing an increase at the onset temperature by ~40 °C and the maximal weight loss rate by 55 °C, which indicates that HNTs entrap and protect the volatile EOs. Krepker et al. [41,44] also showed that the entrapment of carvacrol within HNTs was crucial to achieve increased EO content in LDPE films and high antimicrobial activity. The BET surface area of HNTs, which is 117 m^2^/g, is higher than the BET surface area of bentonite, 59 m^2^/g, and lower than that of O at 194 m^2^/g. HNTs have a hollow structure with hollow cylinders of different diameters and lengths, which renders them a good option to be used as carriers of various chemical substances or additives such as EO components. The clay surface of HNTs consists of silanols and aluminum groups that may interact with other polar groups. Moreover, the large lumen galleries of HNT occupied by air can be loaded with additives. On the other hand, O is characterized by a laminar morphology, with platelets around 1 nm in thickness. The main reactive sites of O are the hydroxyl groups which may promote interactions among the surface of O and other molecules, leading to the intercalation with bioactive molecules in the interlayer space [45].

The results derived from the TGA are in accordance with XRD results where increased *d*-spacing was observed indicating that the C or T molecules were released from the interlayer space of O. Moreover, in the case of B bioactive nanocarriers, lower *d*-spacing was obtained, enhancing the conclusion that C and T molecules were adsorbed on the external surface of B particles and were released at high temperatures above 220 °C.

#### 2.1.3. Antioxidant Activity (AOA)

**Figure 3:**
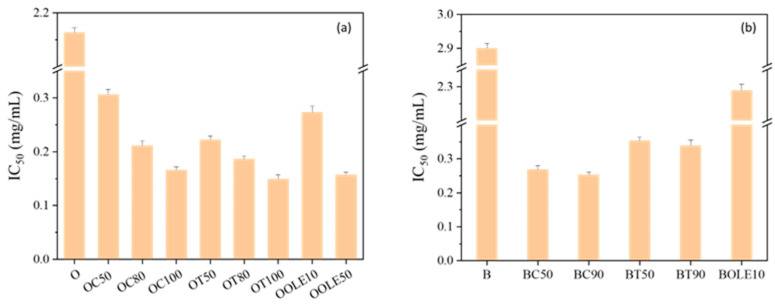
Antioxidant activity of (**a**) organically modified clay nanocarriers (O) and (**b**) bentonite (B) with carvacrol (C), thymol (T) and OLE (OLE).

For the antioxidant activity, among the produced nanocarriers with adsorbed carvacrol (C) and thymol (T), those with *r* = 1:0.5 and above were chosen to be studied. The results of the antioxidant activity of the nanocarriers are presented in Figure 3. The nanocarriers with the highest adsorption percentages show higher antioxidant activity as they have the lowest IC50 value. Among the nanocarriers with olive leaf extract (OLE), those with the ratios *r* = 1:0.1 and 1:0.5 were studied. With the incorporation of C and T in the nanocarriers, their antioxidant activity increased significantly, especially with the increase in the adsorption percentage of the bioactive compounds, compared to that of the nanocarrier without the bioactive compound. Analytically, the organically modified clay (O) with T showed slightly better results compared to the samples with C. This behavior may be attributed to the higher steric hindrance of the phenolic group in thymol, due to the different position of the hydroxyl group on the phenol ring than in carvacrol, which has a significant effect on the antioxidant activity of phenolic compounds [46]. Moreover, when the olive leaf extract was used at a ratio of *r* = 1:0.5 and in combination with the organically modified clay (OOLE50), a slightly higher antioxidant activity was obtained compared to the OC100 sample with *r* = 1:1, and activity was slightly lower compared to the sample OT100 at *r* = 1:1. In bentonite (B) the behavior of the two compounds is reversed. This may be owed to the few hydrogen bonds present due to the lower number of hydroxyl groups that were available only on the surface of bentonite and not in the interlayer space, as described earlier. Moreover, BOLE10 showed a very small increase in antioxidant activity compared to the activity of pure bentonite (B).

#### 2.1.4. Antimicrobial Activity

The antimicrobial activity of bioactive nanocarriers against Gram-negative *E.coli* was investigated. Among the nanocarriers prepared, those with the highest ratio in carvacrol and thymol were selected to be tested for their antimicrobial activity. Consequently, organically modified clay nanocarriers loaded with carvacrol and thymol at the ratios *r* = 1:0.8, 1:0.9 and 1:1 and bentonite nanocarriers loaded with carvacrol or thymol at a ratio of *r* = 1:0.9 were tested.

**Figure 4:**
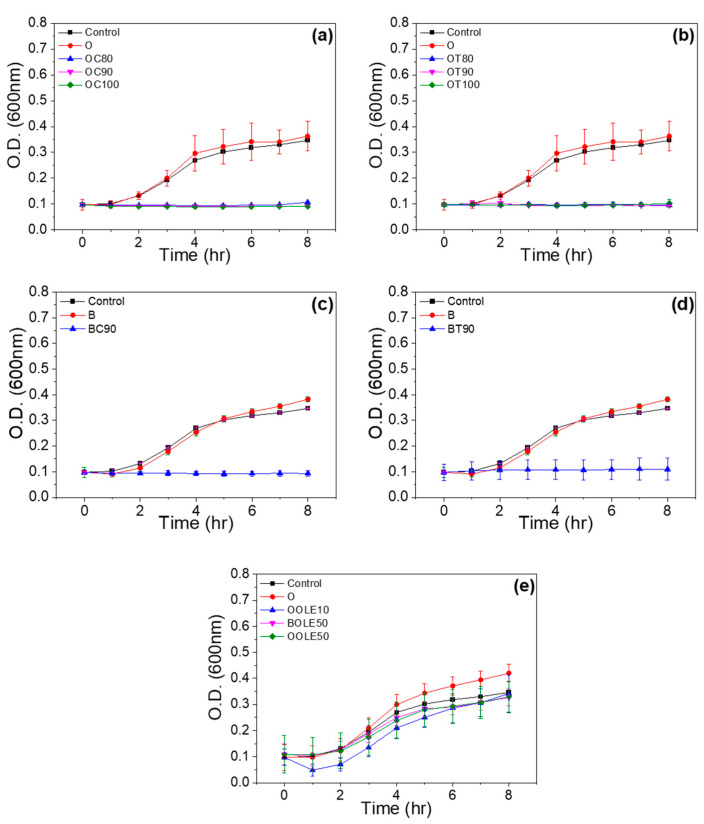
Antimicrobial activity of bioactive nanocarriers: (**a**) carvacrol-loaded organically modified montmorillonite (OC), (**b**) thymol-loaded organically modified montmorillonite (OT), (**c**) carvacrol-loaded bentonite (BC), (**d**) thymol-loaded bentonite (BT) and (**e**) olive leaf extract-loaded organically modified montmorillonite (OOLE) and bentonite (BOLE).

From the bacterial growth curves of Figure 4, we may observe the lag phase, during which no cell growth occurs, and the exponential growth phase where cells start to divide regularly by binary fission and grow by geometric progression. All nanocarriers of organically modified clay with theoretical percentages of carvacrol and thymol at ratios *r* = 1:0.8, 1:0.9 and 1:1 showed complete inhibition of *E. coli* bacterial growth, as shown in the results of Figure 4a,b. In the case of the bentonite nanocarrier, the growth of *E. coli* was inhibited by carvacrol and thymol at a ratio of *r* = 1:0.9 (Figure 4c,d). The bioactive nanocarriers with OLE (Figure 4e) delayed the lag phase and lowered the growth rate and final cell concentration of the microorganism.

The novel materials consisting of adsorbed bioactive compounds onto clay minerals that are presented in this work may control microbial contamination through the reduction of cells’ growth rates and maximum population. Moreover, they lead to the extension of the lag period of the target microorganism, aiming to prolong the product shelf life and maintain its safety [47,48].

### 2.2. Characterization of LDPE Films Loaded with Bioactive Nanocarriers

The bioactive nanocarriers prepared in the present work were dispersed into the LDPE matrix using a mini twin screw extruder to produce masterbatches formed subsequently in films, as described in Section 3. The bioactive nanocarriers chosen to be used were organically modified clay or bentonite with the highest theoretical amount of bioactive substances, carvacrol and thymol (100% theoretical adsorption of bioactive substances on the clay for 96 h). Table 2 presents all the blends’ compositions used for film preparation of LDPE with O or B loaded with the bioactive compounds C, T and OLE. Comparison was made by mixing 5%, 10% and 20% wt. bioactive-O as well as 5% and 10% wt. bioactive-B in LDPE. Films with the use of OLE as bioactive component were obtained by mixing 5% and 10% wt. of bioactive nanocarriers in LDPE. Furthermore, film with assigned name PE_T10 was prepared via direct mixing of thymol (without nanoencapsulation) with LDPE using the mini twin extruder. This sample was used as a control for comparison reasons with the film PE_OT10, which contains an equal amount of thymol encapsulated in the nanocarriers. In the case of films with bentonite and/or OLE, PE-g-MA was used as compatibilizer during blend formation to obtain homogenous films.

#### 2.2.1. XRD Results

The XRD results of all the prepared films are presented in Figure S1. It may be seen that the XRD pattern of LDPE with O indicated a diffraction peak at 2θ of about 2.65°, which shifted to lower 2θ values, between 2.36 and 2.48°, after the incorporation of the bioactive compounds C and T, and to 2.57–2.73° after the incorporation of OLE.

In the case of B, the characteristic reflection 001 occurs at 2θ = 6.82°, as shown from the graphs of LDPE/BOLE films of Figure S1d, and the intensity is weakened. The results agree with the XRD graphs of Figure 2, showing that in the case of films with O, the incorporation of the bioactive compounds takes place between the clay lamellae, whereas in the case of films with B and OLE the bioactive compounds do not enter the interlayer region of the B sheets but are mainly retained on the outer surface of the clay particles.

#### 2.2.2. TGA Results

LDPE films with incorporated bioactive nanocarriers were characterized by TGA measurements. Diagrams and results are presented in Figure S2 and Table S1, respectively. Bioactive substance content in the films after the thermoforming procedure was calculated comparing the TGA curves of the films with and without bioactive substances.

TGA analysis revealed that 0.2–0.3% wt. of carvacrol and thymol remained in the films with 5% OC or OT, while an increase in carvacrol or thymol content was observed at the films incorporated with 10% OC or OT (0.7% wt. carvacrol and 1.2% wt. thymol). Films incorporated with 20% OC or OT presented higher amounts of bioactive substances, 1.6% wt. and 2.3% wt., respectively, after thermoprocessing. Thus, the increase in the bioactive nanocarriers leads to an increase in the final amount of carvacrol or thymol in the films. Films incorporated with 10% wt. OOLE at a ratio of *r* = 1:0.5 presented equal final OLE content after thermoprocessing (1.1% wt.). According to the TGA measurements, 0.9% wt. OLE remained in the films with bentonite initially loaded with 10% wt. BOLE (*r* = 1:0.1).

To provide evidence that the nanoencapsulation process was beneficial for the protection of the bioactive substances during the thermoforming procedure, films with an equal initial amount of thymol as the sample PE_OT10 (i.e., 10% wt.) were also produced via the direct mixing of thymol with LDPE and clay (without nanoencapsulation sample PE_T10). TGA measurements revealed that thymol content in the produced film was 0.1% wt. compared to the respective 1.2% wt. that was found in the films after encapsulation. Similar results were reported by Shemesh et al. [40], who found that less than half of the initial carvacrol content remained in the film after the thermoforming procedure. It is evident from the results, that although loss of bioactive substances occurs during the thermoforming process, the nanoencapsulation procedure is beneficial for the protection of the heat-sensitive bioactive substances.

#### 2.2.3. Mechanical Properties of Films

**Figure 5:**
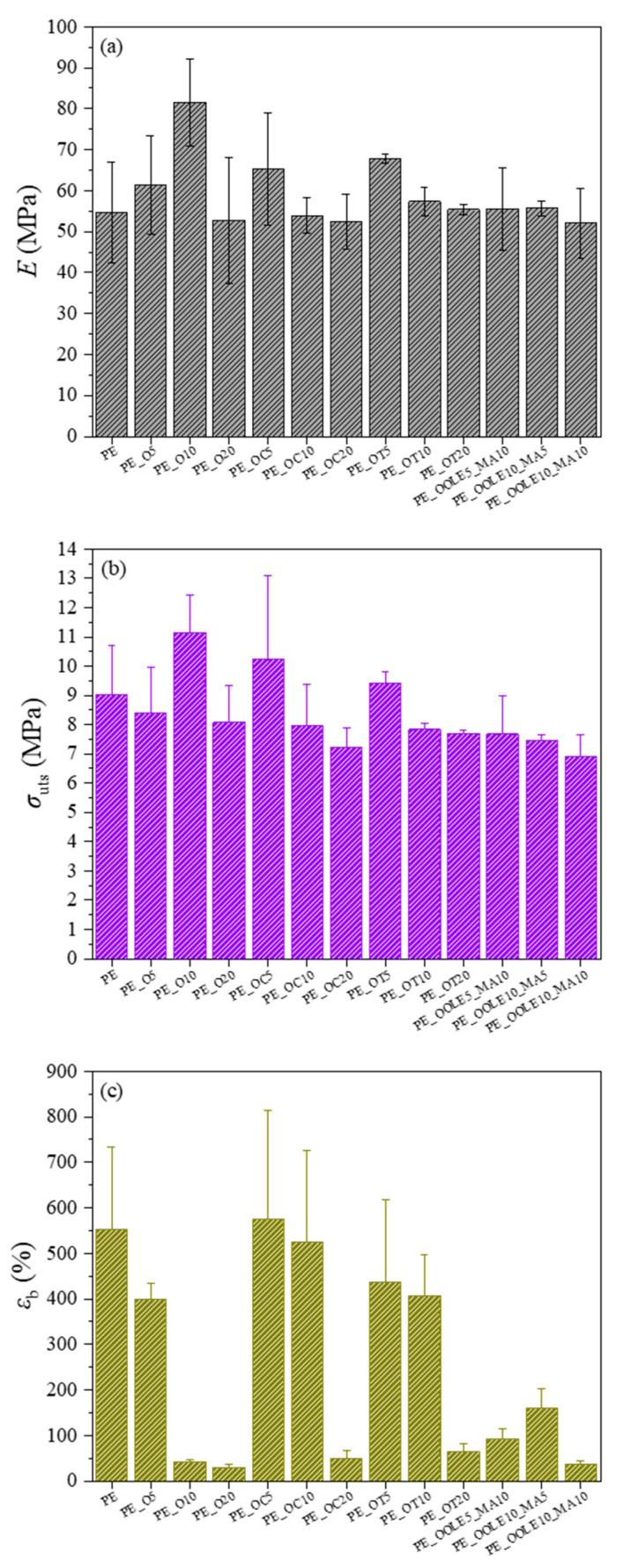
(**a**) Young’s Modulus (*E*), (**b**) tensile strength (*σ*_uts_) and (**c**) strain at break (*ε*_b_) of LDPE films with incorporated organically modified clay nanocarriers (O) with carvacrol (C), thymol (T) and olive leaf extract (OLE).

Mechanical properties provide indispensable information of films’ stiffness, strength and elongation for practical applications [49]. The results of Young’s Modulus (*E*), ultimate strength (*σ*_uts_) and elongation at break (*ε*_b_) obtained for all films are presented in Figure 5a–c, respectively. As shown in Figure 5a, an increase of Young’s modulus values is observed when neat O (5 and 10% wt.) was added to LDPE, compared with unreinforced LDPE films. However, the incorporation of neat O at higher loadings (20% wt.) did not result in any stiffening of neat LDPE films. The tensile strength of the prepared films is only increased after the addition of 10% wt. neat O (Figure 5b), while the strain at break of LDPE films with O generally decreases with increasing amounts of clay (Figure 5c). Maximum reinforcement was achieved after the addition of neat 10% wt. clays, and this behavior can be attributed to the stiffening/strengthening effect of the clay. The results obtained after the addition of 10 and 20% wt. clay in terms of elongation at break are in accordance with other studies published in the literature where lower *ε*_b_ values were detected after the incorporation of clays such as HNT and MT into polymer matrices [50,51,52]. The obtained results can be attributed to the restriction in the chain mobility of LDPE in the presence of clays at low concentrations, which results in higher stiffness and strength and a respective drop in the elongation at break. However, the extensive decrease in the elongation at break without any stiffening/strengthening observed at higher clay loadings, suggests inadequate dispersion and formation of higher number of agglomerates, which act as stress concentration points and result in premature failure. Slightly different trends were observed after the incorporation of clay–bioactive compounds in the neat LDPE films, where the best stiffening/strengthening was achieved after the addition of O loaded with 5% wt. carvacrol or thymol. Stiffness (Figure 5a) and strength values were higher when thymol/carvacrol-based compounds were added in the LDPE films at 5% wt., while the addition of clays loaded with OLE did not result in any reinforcement of LDPE. Films with 10% wt. clay–carvacrol/thymol compounds show lower reinforcing effects compared to their counter parts with 5% wt. loadings, as well as respective films with plain 10% wt. clay. However, the performance of films with 20% wt. clays–bioactive was close to that of respective films with plain 20% wt. clays. Interestingly, the addition of carvacrol and thymol led to a substantial increase in the elongation at break (Figure 5c), with values 12 times higher in the case of PE_OC10 and 10 times higher in PE_OT10, compared to the PE_O10 film. It can be postulated that the interaction of the bioactive compounds with O resulted in lower polarity of the clay–bioactive compound compared to the neat clay, leading to higher compatibility of the compound with the nonpolar LDPE. This facilitated the distribution of the clay–bioactive compound along the LDPE chain leading to higher interfacial interaction between the matrix and the reinforcing element. This beneficial behavior was observed for loading up to 10% wt., while agglomeration prevailed after the addition of excessive amount of clay–bioactive compound (20% wt.). For films of LDPE with O loaded with OLE, the mechanical properties were overall inferior compared to those with 5% wt. C or T, even after the addition of MA at different concentrations. The obtained stiffness and strength were close to those after the addition of 10 and 20% wt. C or T; however, the strain at break was quite low. This suggests that the addition of the MA was not sufficient to overcome the compatibility issues of OLE with LDPE, thus, the agglomerates prevail. Considering the overall performance of the produced films, it may be concluded that the improvement in mechanical properties should be attributed not only to the nanoclay used but also to the interfacial interaction/dispersion of the clay–bioactive compounds in the nanocomposite LDPE films. This means that concentrations of the bioactive compounds higher than 5% wt. did not cause additional development in mechanical properties likely due to the poor dispersion and high surface energy of the clays [53,54].

#### 2.2.4. Antioxidant Activity (AOA)

**Figure 6:**
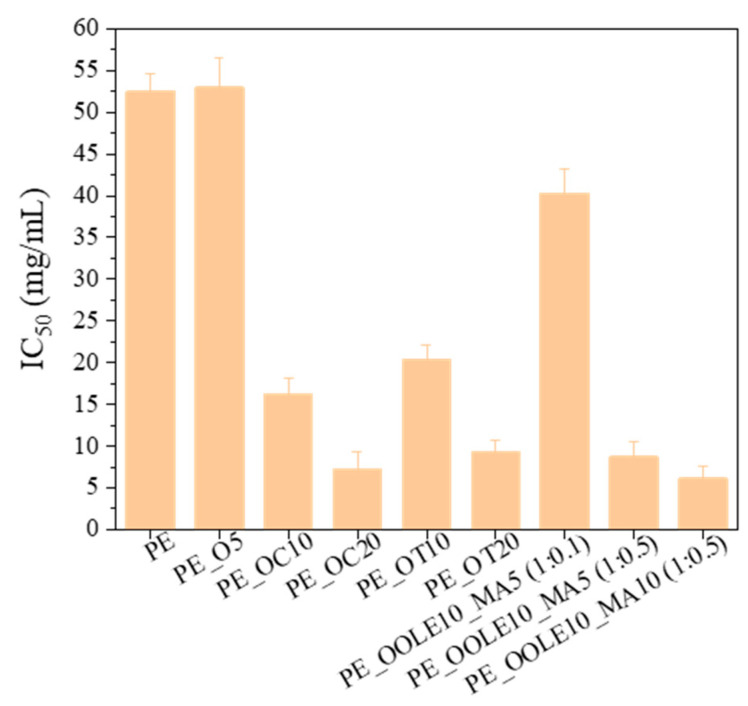
Antioxidant activity of LDPE films with incorporated organically modified clay nanocarriers (O) and bentonite (B) with carvacrol (C), thymol (T) and olive leaf extract (OLE).

In order to determine the ability of bioactive nanocomposite films to control oxidation in foods, their DPPH (2,2-diphenyl-1-picrylhydrazyl) radical scavenging activities were determined. From the results of Figure 6, it may be seen that the films showed significant AOA with the levels ranging from 7.5 to 16.0 mg/mL for PEOC films, and from 9 to 21 mg/mL for PEOT films with the higher AOA appearing in the films with 20% wt. content of organically modified montmorillonite loaded with carvacrol or thymol (PE_OC20 or PE_OT20). Films with OLE presented high AOA in the case of PE_OOLE10_MA5 (1:0.5) and PE_OOLE10_MA10 (1:0.5), whereas the film PE_OOLE10_MA5 (1:0.1) showed low antioxidant activity.

The high antioxidant activity of PE films containing clay with bioactive compounds may be attributed to the phenolic compounds of C, T and OLE which exhibit excellent antioxidant properties. It has been stated in the literature that polyphenols function through mechanisms such as free radical scavenging, single-electron transfer, hydrogen atom transfer and metal chelation [55,56]. Their incorporation into films/coatings is considered as a technique for new approaches to fruit and vegetable preservation since they may lead to moisture loss inhibition, fat, protein, and color oxidation reduction, as well as shelf life extension and food quality improvement [57].

#### 2.2.5. Antimicrobial Activity

**Figure 7:**
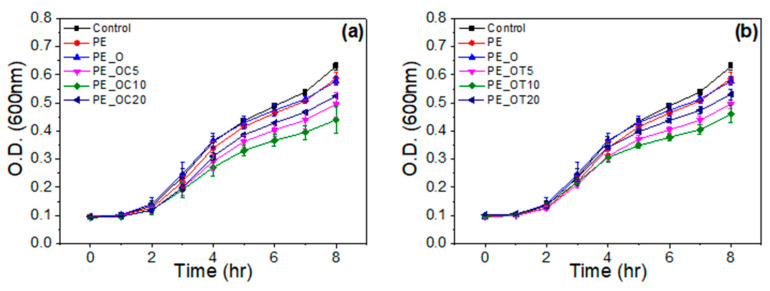
Antimicrobial properties of LDPE films with incorporated organically modified clay nanocarriers (O) with (**a**) carvacrol (C) and (**b**) thymol (T).

As it may be seen from Figure 7, films of LDPE with 5, 10 and 20% wt. content of O loaded with carvacrol and thymol showed an increase in the antimicrobial efficiency compared to films of neat LDPE and LDPE/O. It must be noted that the film with 10% wt. OC and OT presented the highest antimicrobial activity in both cases. The lag time and growth rate of *E. coli* were not affected in this case by the active compounds in films. However, the final cell concentration was reduced compared to the control film. The films presented in this work may control microbial contamination by reducing the maximum growth population, which will result in safer products with a longer shelf life.

## 3. Materials and Methods

### 3.1. Materials

Carvacrol (5-isopropyl-2-methylphenol) 98%, thymol (2-isopropyl-5-methylphenol) ≥ 98.5%, polyethylene-graft-maleic anhydride (PE-g-MA) and DPPH (2,2-diphenyl-1-picrylhydrazyl) were purchased from Sigma-Aldrich (Aldrich, Steinheim, Germany) and used as received. The olive leaf extract (OLE) was prepared according to the method described by Chatzikonstantinou et al. [58]. The organically modified montmorillonite (O) Nanomer^®^ I.44P, a surface-modified montmorillonite clay with 40 % wt. dimethyl dialkyl (C14–C18) amine, was produced by Nanocor Inc. (Hoffman Estates, IL, USA), supplied by Sigma-Aldrich (St. Louis, MO, USA) and used without further treatment. Nanoclay, hydrophilic bentonite was provided from Sigma-Aldrich (Aldrich, Steinheim, Germany). The solvent absolute ethanol was purchased from Merck (Merck KGaA, Darmstadt, Germany). Low-density polyethylene (DOW™ LDPE 352E) was kindly provided from Achaika Plastics S.A. (Aigio, Greece).

### 3.2. Preparation of Bioactive Nanocarriers

**Figure 8:**
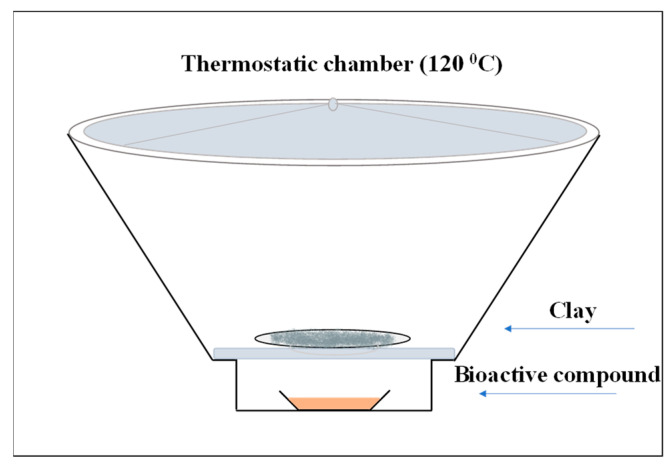
Adsorption method for the preparation of bioactive nanocarriers (organically modified montmorillonite or bentonite loaded with the bioactive compounds carvacrol and thymol).

For the incorporation of carvacrol (C) and thymol (T) into the organically modified montmorillonite (O) and bentonite (B), the adsorption method was applied. The O and B were dried at 120 °C for 24 h prior to use, to remove the adsorbed moisture. For each experiment, the appropriate quantity of the bioactive compound was placed on a glass plate and inserted at the bottom of a closed and heated chamber. Then, the corresponding quantity of clay, 3 g, was added on a glass plate and placed in a position higher than the bioactive compound. The chamber was sealed and left for 96 h at 120 °C, where intra-layer and surface adsorption of the bioactive compounds on O and B took place. The schematic representation of the system used for the adsorption of C and T into the O or B is presented in Figure 8. The incorporation of olive leaf extract (OLE) into O and B was applied through the solution method, in which an aqueous suspension was prepared in the presence of the clay and the extract and stirred for 24 h at ambient temperature. The suspension was then placed in an oven at 50 °C until the water evaporated and the final product was collected.

### 3.3. LDPE/Clay Bioactive Nanocomposite Film Preparation

LDPE and bioactive nanocarriers (O or B loaded with the bioactive compounds) were melt-compounded, using a minilab twin co-rotating extruder (Haake Mini Lab II, ThermoScientific, ANTISEL S.A., Athens, Greece). The operating temperature was 160 °C, the screw speed was 100 rpm and the total processing time was 10 min. The obtained masterbatch was pressed with a Manual Hydraulic Press with heated platens (Specac, ANTISEL S.A., Athens, Greece) between two Teflon sheets at 120 °C and 70 bar pressure for 2 min and rapidly quenched in an ice-water bath for film formation.

### 3.4. Characterization Techniques

#### 3.4.1. Structural Characterization Using X-ray Diffraction (XRD)

The characterization of the obtained films using XRD, in which the incorporation of the bioactive compounds into the interlayer space of nanocarrier was confirmed, was performed on an advanced Brüker D8 diffractometer (Bruker, Analytical Instruments, S.A., Athens, Greece) with CuKa radiation (λ = 1.541874 Å). The diffractometer was operated at constant temperature (20 °C) and the monochromatic beam was under constant voltage and current, 40 kV and 40 mA, respectively. The distance *d* between the nanocarrier sheets was calculated from the 001 reflectance. The scanning parameters were set as follows: 2θ range 2–20°, increment 0.03°, PSD 0.764 and slit width 0.6 mm. Each sample was carefully placed on glass sample holders so that its surface was at the same level as the reference plane of the instrument.

#### 3.4.2. Thermogravimetric Analysis (TGA)

TGA was carried out in Pt-Rh crucibles in a Pyris 1 TGA Netzsch STA 449 C Jupiter (Netzsch, Selb, Germany) thermal analyzer under nitrogen and at a heating rate of 20 °C/min. The temperature accuracy of the instrument was < 1 °C.

#### 3.4.3. Mechanical Analysis

Tensile tests were performed using a universal testing machine, the Simantzu AX-G equipped with a 5KN load cell (Simantzu, Asteriadis, S.A., Athens, Greece) according to the American Society for Testing and Materials (ASTM) D638. Three to five dog-bone type V specimens of each film were tested at a deformation rate of 30 mm/min. Young’s modulus, tensile strength and elongation at break were calculated. Statistical calculations (mean values and standard deviation) were performed on the results of three to five specimens.

### 3.5. Determination of Antioxidant Activity Based on the Free Radical Binding Capacity of DPPH

The DPPH (2,2-diphenyl-1-picrylhydrazyl) free-radical scavenging method was used for the determination of the antioxidant activity of the samples [59,60,61], in which the bleaching rate of a stable free radical, DPPH•, is monitored at a characteristic wavelength in the presence of the sample. First, an ethanolic sample solution (1 mg/mL) and an ethanolic solution of DPPH (1 mM) were prepared. Appropriate volumes of ethanol and ethanolic sample solution as well as 400 μL of EtOH-DPPH solution were added up to a final volume of 4 mL. The samples were kept at room temperature (RT) for 30 min in the dark. The control solution consisted of ethanol and DPPH, and its absorbance was measured at 517 nm. All measurements were carried out in duplicate, and the results are presented as the mean. The antioxidant activity (AOA) is expressed as percentage of inhibition according to the following equation: AOA% = 100 − [((A_sample − A_blank)∙100)/A_control], where A_blank is the EtOH-sample adsorption at 517 nm and A_control is the EtOH-DPPH adsorption at 517 nm.

### 3.6. Determination of Antimicrobial Activity

#### 3.6.1. Bacterial Culture Preparation

The Gram-negative *Escherichia coli* (*E. coli*) BL21DE3 microorganism was used to test the antimicrobial activity of the films. The strains used were from our lab collection and the Health Protection Agency, Porton Down, Salisbury, UK. From the stock bacterial population of *E. coli* BL21DE3 strain, 100 μL of the bacterial population was initially inoculated into 5 mL of fresh Lysogeny Broth (LB) and incubated overnight (O/N) at 37 °C under continuous stirring at 180 rpm.

#### 3.6.2. Bacterial Reduction Assay

The next day, the 600 nm absorbance of the pre-culture was measured and diluted with the appropriate amount of fresh LB so that the optical absorbance of the new culture was 0.08. This new culture was then re-incubated at 37 °C with continuous stirring at 180 rpm until the bacterial population reached an optical absorbance of 0.2–0.5. The culture was then centrifuged at 4000 rpm for 5 min, the supernatant was removed and the precipitate was redissolved in serum (0.9 % *w*/*v* NaCl) of equal volume. After three successive washes, samples of a bacterial population of 10^7^ CFU/mL were prepared by dilution. Then, 0.5 mg of each sample was placed in an Eppendorf tube containing 100 μL of the bacterial population. The control consisted of 100 µL of bacteria in the absence of sample. The Eppendorf tubes were placed in a cold chamber for 12 h in the case of nanocarriers and for 36 h in the case of LDPE films loaded with bioactive nanocarriers. The difference in the time interval is due to the extended release time of the bioactive substances from the films. After the above-mentioned time interval, 25 μL of the bacterial population interacting with the sample was inoculated into 225 μL of fresh LB in an ELISA microplate. The microplate was placed in a chamber for incubation at 37 °C under continuous stirring. Every hour for a total of 8 h the optical absorbance of the microplate at 600 nm was measured.

## 4. Conclusions

The novelty of the work consists of the successful incorporation of active volatile compounds into unmodified and organo-modified montmorillonite through a green methodology and their further incorporation in LDPE, providing films with controlled antioxidant and antimicrobial activity that are promising candidates for active packaging films for fresh fruits and vegetables. Moreover, the incorporation of olive leaf extract into clays and subsequently in LDPE is reported for the first time.

Τhis study showed that the direct incorporation of carvacrol and thymol onto the surface and interlayer of nanoclays and their subsequent mixing with LDPE resulted in films with high thermal stability and improved mechanical properties. Moreover, the advantages of the prior encapsulation of bioactive compounds onto clays was the pronounced antioxidant activity of the films, which may be owed to the prevention of volatilization of thymol, carvacrol and olive leaf extract. This is very important for the application of these films during food formulations with improved oxidative stability. The produced films may control microbial contamination by reducing the maximum growth population which will result in safe products with a long shelf life.

The present study demonstrates the contribution of bioactive compounds to the properties of the produced bioactive nanocarriers, depending on the incorporation rate and the characteristics of the nanostructures. On this basis, LDPE films containing clay-bioactive nanocarriers at levels of 5–20% wt. could be excellent candidates for use as a desirable antioxidant/antimicrobial packaging material with enhanced thermal and mechanical properties. The characterization results showed that the incorporation of carvacrol and thymol into the interlayer region of the organically modified clay was achieved, in contrast to inorganic bentonite where bioactive compounds were mainly retained on the outer surface of clay particles. For the nanocarriers with incorporated olive leaf extract, the results were indicative of achieving an exfoliated structure of the clay. Regarding the thermal stability of the bioactive nanocarriers, the results demonstrated a protective effect of the clay against the thermal loss of the bioactive compounds. The antioxidant activity of the nanocarriers after incorporation of the bioactive compounds increased significantly, especially with increasing incorporation rate. Additionally, the nanocarriers of organically modified clay and bentonite with the highest incorporation rates of carvacrol and thymol showed complete inhibition of *E. coli* bacterial growth.

The antioxidant activity of the LDPE films with loaded bioactive nanocarriers indicated increased antioxidant activity with 20% wt. of organically modified montmorillonite with carvacrol (OC) or thymol (OT) at the clay:bioactive substance ratio 1:1, and 10% wt. of organically modified montmorillonite with olive leaf extract (OOLE) at a ratio of 1:0.5. Films with 10% OC and OT presented the highest antimicrobial activity in both cases. 

The future aim of this novel film formation is to be used as the interlayer of a scaled-up three-layered membrane that will act as a “reservoir” of bioactive substances with controlled release and long-term durability.

## Figures and Tables

**Table 1:** XRD results and thermal properties of neat clay and bioactive nanocarriers.

Material’s Code Name	Clay:Bioactive Substance Ratio (*r*)	2θ (^ο^)	*d*_001_ (Å)	TGA Results	
				*T*_20_ (°C)	Βioactive Substance Content (% wt.) ^2^
O		3.54	25	362	-
	O/C hybrids				-
OC1	1:0.01	3.44	25.7	-	-
OC10	1:0.1	2.58	34.2	-	-
OC50	1:0.5	2.40	36.8	297	14
OC70	1:0.7	2.40	36.8	-	-
OC80	1:0.8	2.48	35.6	280	24
OC90	1:0.9	2.40	36.8	244	27
OC100	1:1	2.37	37.3	240	29
	O/T hybrids				
OT1	1:0.01	3.43	25.8	-	-
OT10	1:0.1	2.65	33.3	-	-
OT50	1:0.5	2.40	36.8	238	19
OT70	1:0.7	2.43	36.4	-	-
OT80	1:0.8	2.52	35.1	288	23
OT90	1:0.9	2.40	36.8	203	25
OT100	1:1	2.41	36.7	202	26
	O/OLE hybrids				
OOLE10	1:0.1	3.35	26.4	349	4
OOLE50	1:0.5	n.d.	n.d.	297	9
B		6.80	13	n.d.^1^	-
	B/C hybrids				
BC50	1:0.5	7.20	12.3	223	15
BC70	1:0.7	7.28	12.1	-	-
BC90	1:0.9	7.34	12	198	17
	B/T hybrids				
BT50	1:0.5	7.10	12.5	331	13
BT70	1:0.7	7.20	12.3	-	-
BT90	1:0.9	7.25	12.2	153	20
	B/OLE hybrids				
BOLE10	1:0.1	6.40	13.8	n.d.^1^	3
BOLE50	1:0.5	n.d.	n.d.	346	18

^1^ n.d.: not detected; ^2^ calculated from TGA.

**Table 2:** Composition of blends for LDPE film preparation.

Film’s Code Name	Blends	Clay:Bioactive Substance Ratio (*r*)	Composition (% wt.)
PE_O5	LDPE/O	-	95/5
PE_O10	LDPE/O	-	90/10
PE_O20	LDPE/O	-	80/20
PE_OC5	LDPE/OC	1:1	95/5
PE_OC10	LDPE/OC	1:1	90/10
PE_OC20	LDPE/OC	1:1	80/20
PE_OΤ5	LDPE/OT	1:1	95/5
PE_OΤ10	LDPE/OT	1:1	90/10
PE_T10	LDPE/T	-	90/10
PE_OΤ20	LDPE/OT	1:1	80/20
PE_OOLE5_MA5	LDPE/OOLE/PE-g-MA	1:0.1	90/5/5
PE_OOLE5_MA10	LDPE/OOLE/PE-g-MA	1:0.5	85/5/10
PE_OOLE10_MA5	LDPE/OOLE/PE-g-MA	1:0.1	85/10/5
PE_OOLE10_MA10	LDPE/OOLE/PE-g-MA	1:0.5	80/10/10
PE_BC5_MA10	LDPE/BC/PE-g-MA	1:0.9	85/5/10
PE_BC10_MA10	LDPE/BC/PE-g-MA	1:0.9	80/10/10
PE_BT5_MA10	LDPE/BT/PE-g-MA	1:0.9	85/5/10
PE_BT10_MA10	LDPE/BT/PE-g-MA	1:0.9	80/10/10
PE_BOLE5_MA5	LDPE/BOLE/PE-g-MA	1:0.1	90/5/5
PE_BOLE10_MA5	LDPE/BOLE/PE-g-MA	1:0.1	85/10/5

## Data Availability

Not applicable.

## Supplementary Materials

The following supporting information can be downloaded at https://www.mdpi.com/article/10.3390/molecules28072945/s1, Figure S1. XRD patterns of LDPE films with organically modified montmorillonite loaded with (a) carvacrol (OC), (b) thymol (OT) and (c) olive leave extract (OOLE) and (d) bentonite loaded with olive leave extract (BOLE); Figure S2. TGA profiles of films incorporated with bioactive nanocarriers with (a) carvacrol, (b) thymol and (c) OLE; Table S1. Composition of selected films based on TGA analysis.

## Sample Availability

Samples of the compounds are not available from the authors.
