# Supplementary material for: Designing Antioxidant and Antimicrobial Polyethylene Films with Bioactive Compounds/Clay Nanohybrids for Potential Packaging Applications
Source: Molecules. 2023 Mar 25;28(7):2945. doi: 10.3390/molecules28072945 (PMC10095763; doi:10.3390/molecules28072945)
Supplement: Supplementary file 1 [file molecules-28-02945-s001.zip › molecules-2242311-supplementary.pdf]

molecules

MDPI

Supplementary Material

Designing antioxidant and antimicrobial polyethylene films with bioactive compounds/clay nanohybrids for potential packaging applications

Konstantinos Safakas[1], Iro Giotopoulou[2], Archontoula Giannakopoulou[3], Katerina Katerinopoulou[1], Georgia C. Lainioti[1,*], Haralambos Stamatis[3], Nektaria-Marianthi Barkoula[2] and Athanasios Ladavos[1,*]

1 Department of Food Science & Technology, University of Patras, GR-30100, Agrinio, Greece; ksafakas@upatras.gr (K.S.), akaterin@upatras.gr (K.K.), glainioti@upatras.gr (G.C.L.), alantavo@upatras.gr (A.L.)
2 Department of Materials Science and Engineering, University of Ioannina, GR-45110, Ioannina, Greece; i.giotopoulou@uoi.gr (I.G.), nbarkoul@uoi.gr (N.-M.B.)
3 Department of Biological Applications and Technology, University of Ioannina, GR-45110, Ioannina, Greece; a.giannakopoulou@uoi.gr (A.G.), hstamati@uoi.gr (H.S.)
* Correspondence: glainioti@upatras.gr (G.C.L.), alantavo@upatras.gr (A.L.); Tel.: (+30 26410 74134; +30 26410 74126)

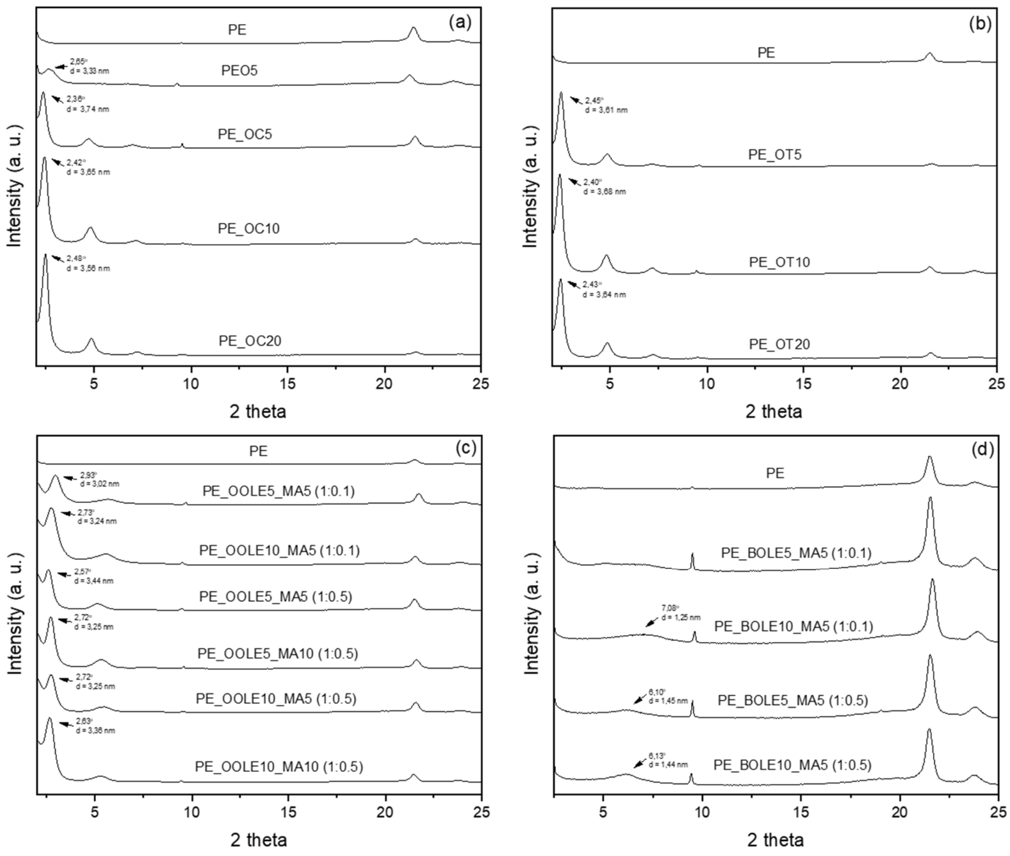

Figure S1. XRD patterns of LDPE films with organically modified montmorillonite loaded with (a) carvacrol (OC) (b) thymol (OT), (c) solgar (OOLE) and (d) bentonite loaded with solgar (BOLE).

molecules

MDPI

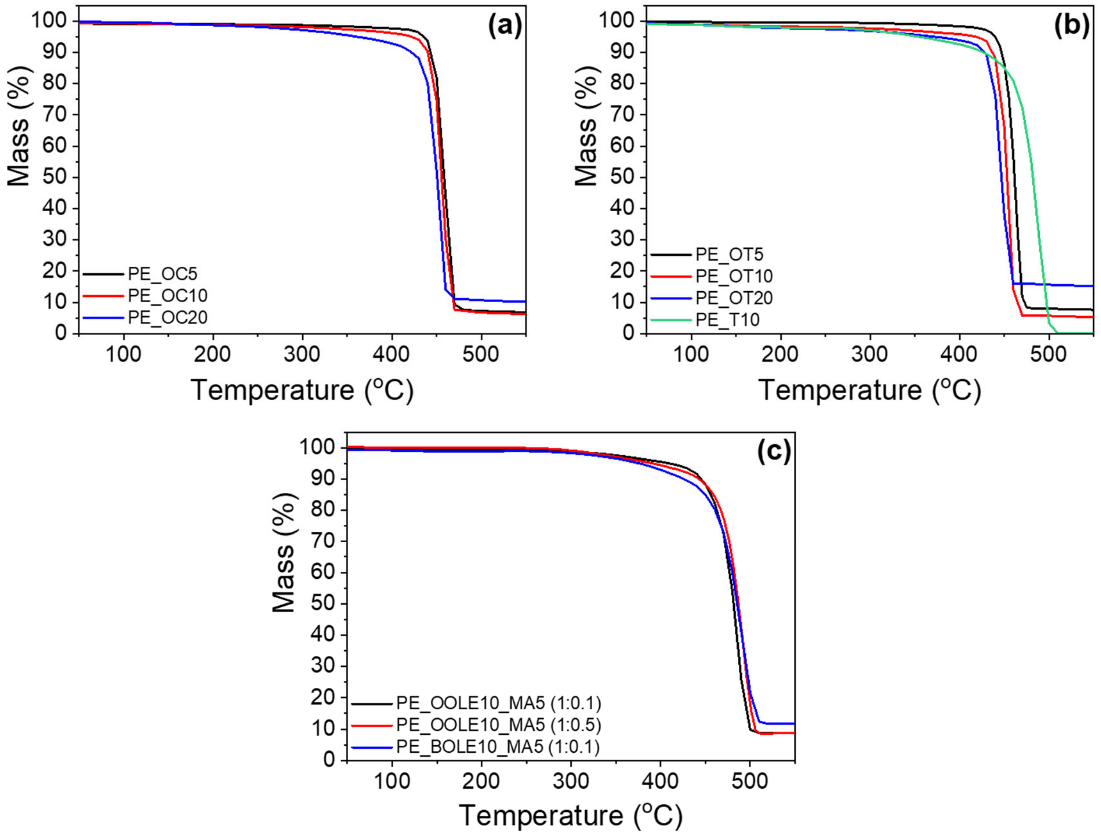

Figure S2. TGA profiles of films incorporated with bioactive nanocarriers with (a) carvacrol, (b) thymol and (c) OLE.

Table S1. Composition of selected films based on TGA analysis.

Film's code name	Blends	Clay:bioactive substance ratio (*r*)	Composition (% wt.)	Bioactive substance content (% wt.)
PE_OC5	LDPE/OC	1:1	95/5	0.2
PE_OC10	LDPE/OC	1:1	90/10	0.7
PE_OC20	LDPE/OC	1:1	80/20	1.6
PE_OT5	LDPE/OT	1:1	95/5	0.3
PE_OT10	LDPE/OT	1:1	90/10	1.2
PE_T10	LDPE/T	-	90/10	0.1
PE_OT20	LDPE/OT	1:1	80/20	2.3
PE_OOLE10_MA5	LDPE/OOLE/PE-g-MA	1:0.1	85/10/5	1.1
PE_OOLE10_MA5	LDPE/OOLE/PE-g-MA	1:0.5	85/10/5	1.1
PE_BOLE10_MA5	LDPE/BOLE/PE-g-MA	1:0.1	85/10/5	0.9
